# Is emphysema a risk factor for pneumothorax in CT-guided lung biopsy?

**DOI:** 10.1186/2193-1801-2-196

**Published:** 2013-04-30

**Authors:** Nobuhiro Asai, Yasutaka Kawamura, Ikuo Yamazaki, Keiji Sogawa, Yoshihiro Ohkuni, Toshihiro O’uchi, Akihito Kubo, Etsuro Yamaguchi, Norihiro Kaneko

**Affiliations:** Department of Pulmonology, Kameda Medical Center, Zip296-8602 929 higashi-cho, kamogawa-city, Chiba, Japan; Department of Radiology, Kameda Medical Center, Chiba, Japan; Department of Internal Medicine, Division of Respiratory Medicine and Allergology, Aichi Medical University School of Medicine, Aichi, Japan

**Keywords:** CT-guided percutaneous lung biopsy, Pneumothorax, Emphysema

## Abstract

**Introduction:**

Computed tomography (CT)-guided lung biopsy is commonly used to make a histological diagnosis for pulmonary lesions. Its most common complication is pneumothorax. While it is thought that CT-guided lung biopsy should be avoided in patients with emphysema, however, there is no scientific report documenting the relationship the occurrence of pneumothorax and the severity of emphysema.

**Purpose and methods:**

To investigate the relationship between the severity of emphysema and the frequency of pneumothorax, we retrospectively reviewed all the patients who received CT-guided lung biopsy. Severity of emphysema is evaluated by Goddard classification, a visual scale by which areas of vascular disruption and low attenuation value were scored for each lung field of high resolution CT.

Patients’ characteristics, prognostic accuracy of this method, size and location of the lesion, length of intrapulmonary biopsy paths, and frequency of complications such as pneumothorax or intrapulmonary hemorrhage were evaluated.

**Results:**

One hundred-two patients (69 males and 33 females) received 102 procedures. Diagnostic accuracy was 90.2%. Pneumothorax occurred in 41 of 102 biopsies (40.2%). Chest tube placement was required in 3 out of the 41 cases (7.3%) complicated by pneumothorax (2.9% of all the biopsies). The longer lesion depths from pleura were, the more frequently pneumothorax occurred (6.67 vs 3.66 mm, p=0.019). No correlation was found between location of lesions and frequency of pneumothorax. No significant differences of COPD staging or LAA score were seen between the patients with and without pneumothorax (5.73 vs 4.32 points, p=0.339).

**Conclusion:**

We suggest that severity of emphysema such as stage I or II COPD may not be related to the frequency of pneumothorax.

## Introduction

Computed tomography (CT)-guided lung biopsy is commonly used to make a histological diagnosis for pulmonary lesions (Harter et al. 1983;Li et al. 1996;Klein & Zarka 1997). The diagnostic accuracy of this method has been reported and ranges from 64-97% (Klein & Zarka 1997;Hiraki et al. 2009;Tomiyama et al. 2006). In general, CT-guided lung biopsy is regarded as a safe procedure, although some serious complications such as cardiac tamponade, empyema, air embolism or seeding of malignant cells are rarely experienced (Sinner 1976;Ibukuro et al. 2009;Perlmutt et al. 1989). The most common complication related to CT-guided lung biopsy is pneumothorax, followed by hemorrhage. The rate of pneumothorax reported varies widely and ranges from 8-69%, depending on location or size of the lesion, needle used, as well an experience of the operator (Li et al. 1996;Klein & Zarka 1997;Hiraki et al. 2009;Tomiyama et al. 2006;Perlmutt et al. 1989;Khan et al. 2008).

Some physicians and statements have demonstrated that emphysema is a risk factor for pneumothorax (Tomiyama et al. 2006;National Institute of Health, National Heart, Lung, and Blood Institute Global Initiative for Chronic Obstructive Lung Disease 2001;Consensus of Percutaneous Lung Needle Biopsy 2007). Actually, physicians are reluctant to perform CT-guided lung biopsy in some cases with outstanding emphysema revealed by chest CT hesitating to perform it because severe pneumothorax needing chest tube drainage is likely to occur. Actually, we hesitate as others would in performing CT-guided lung biopsy in cases in which we consider that severe pneumothorax requiring a chest tube drainage would likely occur. Although transbronchial lung biopsy (TBLB) is commonly used in diagnosing lung cancer, it is often not sufficient to make a diagnosis of peripheral or small lesions. Currently, TBLB with concurrent navigation systems shows a high accuracy in diagnosis of peripheral and small lesions compared with conventional methods (Arenberg 2009;Makris et al. 2007;Gildea et al. 2006). However, it does not make CT-guided lung biopsy unnecessary in terms of a diagnostic accuracy. While the notion that emphysema is a risk factor for pneumothorax is thought to be reasonable among physicians, the correlation between the rate of pneumothorax and emphysema has never previously been reported. For the purpose of examining the relationship between the frequency of pneumothorax and the severity of emphysema using the Goddard classification which reflects the severity of COPD, we retrospectively analyzed all the patients who underwent CT-guided needle biopsy at our institution from January 2006 to April 2011. This is the first report demonstrating the correlation of the frequency of pneumothorax with the severity of emphysema.

## Patients and methods

We had sought and obtained approval from our institute to use patients’ records for this study. All patients’ confidentiality was maintained.

### Eligibility

All patients who underwent CT-guided lung biopsy in our institution from April 2006 to August 2011 were eligible in this study. One hundred four CT-guided lung biopsies were performed in 102 patients. Informed consent was obtained before the procedure in all cases. All the examinations were performed strictly based on clinical indications alone.

The decision for performing lung biopsy was made in the following cases:

The indication for the lung biopsy was made as follows.

1. Suspected malignancy on the basis of CT morphologic criterion such as shape and margination.
2. Negative bronchofiberopic examination with any reasons.
3. Lesion initially classified as benign but showed increase in size during follow-up examination.
4. Lesion initially regarded as benign or inflammatory but did not respond to therapy.

One to three biopsy procedures were done per time depending on specimens obtained or on whether complication occurred or not by the procedure. Patients’ background, prognostic accuracy of this method, size and location of the lesion, length of intrapulmonary biopsy paths, frequency of complications such as pneumothorax or intrapulmonary hemorrhage were evaluated. In addition, the frequency of pneumothorax and chest tube placement were analyzed in terms of the scores by Goddard classification or severity of chronic obstructive pulmonary disease (COPD) as well as various variables such as size and location of lesion, length between the lesion and the thoracic wall, and whether interstitial pneumonia exit or not.

### Procedure

Patients were placed in the prone, spine, or lateral decubitus positions, depending on the location of the lesion to provide the shortest route for lung biopsy. Biopsies were performed to avoid ribs, bullae, vessels and fissures. Normal platelet counts and coagulation parameters such as prothrombin time or activated prothrombin time of all the patients were confirmed within 3 days before the procedure and made sure that they had taken no anticoagulants in the previous 1-2 weeks. At the site of puncture, 10ml 1% xylocain was administered subcutaneously as local anesthesia. After skin incision, all biopsies were performed with an 18-gauge coaxial needle system. The needle was manually pushed into the lung lesion and biopsy was repeated in the same session. Specimens obtained were immediately immersed in 10% formalin solution and sent for examination to the staff pathologist of our hospital. After the procedure, a chest CT scan was routinely done to detect pneumothorax or intrapulmonary hemorrhage. Patients with asymptomatic pneumothorax or intrapulmonary hemorrhage were treated conservatively with monitoring of vital signs and follow-up chest radiographs to confirm stability. A chest tube was inserted for drainage in patients who had pneumothorax with signs of respiratory distress or shortness of breath. All the patients were admitted to the hospital and discharged on the next day in an uneventful course.

### Evaluation of COPD

**Figure 1:**
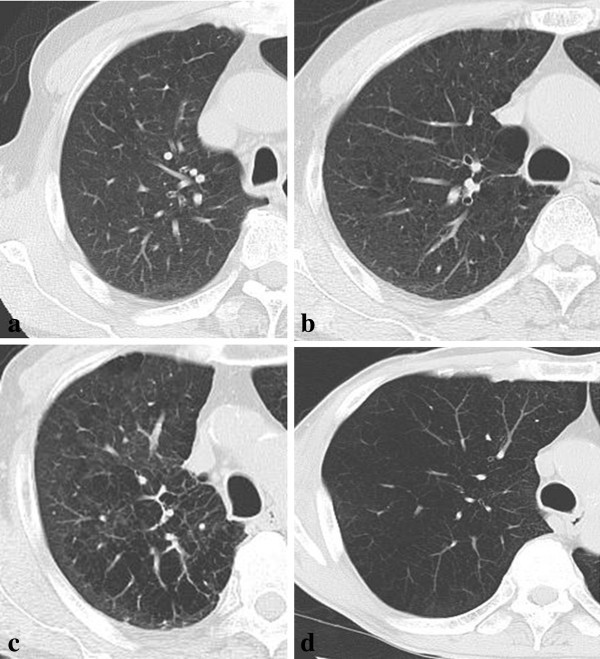
**Shows the relationship between LAAs and the scores of Goddard classification. a**. showing 0 to 25 % of LAA in the lung, Goddard classification 1. **b**. showing 25 to 50 % of LAA in the lung, Goddard classification 2. **c**. showing 50 to 75 % of LAA in the lung, Goddard classification 3. **d**. showing 75 to 100 % of LAA in the lung, Goddard classification 4.

High resolution computed tomography (HRCT) was performed for all the patients to detect low attenuation area (LAA) before CT-guided lung biopsy. Severity of COPD is evaluated by Goddard classification Goddard et al. (1982), which is a visual scale that area of vascular disruption and low attenuation value were scored for each lung field. Zero represented no abnormality; 1 was given for up to 25%, 2 for up to 50%, 3 for up to 75%, and 4 for total involvement or almost total absence of normal lung tissue. There was a possible score of 24 as maximum for each patient. HRCT was assessed by a pulmonologist and a radiologist (Figure 1).

### Statistical analysis

Statistical analysis was examined using Statview. Differences were considered significant when *P* value was less than 0.05.

## Results

This study included 69 male (67.6%) and 33 female (32.4%). The mean age of the population was 69.9 years (range, 42 to 86 years). The mean size of the lesions was 28.8 mm (range 10 to 85 mm). The lesion was in the upper and middle lung field in 55 patients (53.9%) and in the lower lung field in 47 patients (46.1%). The mean length from pleura to the lesion was 5.6 mm (SD±9.6, range 0 to 46.6 mm). In terms of scores by Goddard classification, the average point was 5.2 (SD±4.9, range 0 to 24). Table 1 shows variables in 102 patients classified into two groups: those with and without pneumothorax.

**Table 1:** **Relationship between occurrence of pneumothorax and clinical factors evaluated.**

Clinical factors	Patients with pneumothorax	Patients without pneumothorax	***p***value
No. (%)	41 (40.2%)	61 (59.8%)	NS
Age, yr	72.3 (55-84)	68.3 (42-86)	NS
Male/Female	30/11	39/22	NS
Location of lesion			
Upper or middle	22	33	NS
Lower	19	28	
Lesion size, mm	23.6 (10-65.8)	32.3 (10-85)	0.006
Lesion depth, mm	6.67 (0-46.6)	3.66 (0-39)	0.019
COPD staging			
I	17	17	0.199
II	8	4	0.062
III	1	0	0.402
IV	0	0	1.000
Unknown	5	15	0.137
LAA (±SD)	5.73 (±5.6)	4.79 (±4.32)	0.339
LAA in puncture route (±SD)	0.73 (±0.95)	0.77 (±0.86)	0.831

Data are presented as mean (range) or No. LAA was scored by Goddard classification.

Pneumothorax occurred in 41 of 102 biopsies (40.2%). Chest tube placement was required in 3 out of the 41 cases (7.3%) complicated by pneumothorax (2.9% of all biopsies). The mean size of the lesions in the patients with pneumothorax was smaller than those without (*p*=0.006). The longer lesion depths from pleura were, the more frequently pneumothorax occurred as shown in Table 2 (*p*=0.019), while no statistical significance was seen. No correlation was found between location of lesions and frequency of pneumothorax. Forty-seven of the 102 patients (46.1%) were diagnosed as COPD by spirometry. Forty-six of the 47 patients (97.9%) were classified as COPD I and II. Twenty patients (19.6%) did not undergo pulmonary function test although they have a heavy smoking history. Thirty-five of the 82 patients (42.7%) did not fulfill the criteria of COPD by the result of the pulmonary function test. No significant differences of COPD staging or LAA were seen between the patients with and without pneumothorax. In terms of Goddard Classification scores for the CT slices of puncture tract, there was no significant difference in the two groups.

**Table 2:** **Relationship between frequency of pneumothorax and lesion depth from pleura.**

Lesion depth (mm)	n	Pneumothorax	Pneumothrax requiring chest tube	Pneumothorax rate (%)
0	57	15	0	26.3
< 10	24	14	1	58.3
10-15	6	3	0	50
15 <	15	9	2	60

### Diagnostic yield

The specimens obtained from the 102 lesions were adequate for diagnosis. The diagnostic yields for each lesion size and overall are shown in Table 3. Non-diagnostic results were obtained in 10 of the 102 lesions. Malignant and benign diagnosis were obtained by biopsy in 72 and 21 lesions, respectively. A final diagnosis of malignancy was made in 77 lesions. These malignant diagnoses were confirmed by the surgical specimens (n=72), other examinations including lymph node biopsy (n=1) and ultrasoundsonography-guided lung biopsy (n=3), or obvious post procedural malignant processes (n=1). A final diagnosis of benign disease was made in 25 lesions and based on the characteristics of the surgical specimen (n=2), lesion regression with conservative or medical therapy (n=2), or stable lesion size for at least 1 years (n=21). A final diagnosis of benign disease was made in 25 lesions. They were confirmed by microscopic findings of the surgical specimen (n=2), lesion regression with conservative or medical therapy (n=2), or stable lesion size for at least 1 years (n=21).

**Table 3:** **Diagnostic yield of CT-guided percutaneous lung biopsy**

Diagnostic Yield	≦10mm (n=2)	10-20mm (n=28)	20-30mm (n=37)	30mm≦ (n=35)	Overall (n=102)
True-positive results	0	16	24	28	68
True-negative results	1	7	8	6	22
False-positive results	0	0	0	0	0
False-negative results	0	0	2	0	2
Nondiagnostic results	1	5	3	1	10

The overall sensitivity, specificity, positive predictive value and negative predictive value for the diagnosis of malignancy were 89.5% (68 of 76 lesions), 88% (22 of 25 lesions), 100% (68 of 68 lesions) and 75.6% (25 of 33 lesions), respectively. The diagnostic accuracy was 90.2% (92 of 102 lesions).

## Discussion

It has been reported that 30 % of elderly patients who are more than 65 years with lung cancer are complicated with COPD (van de Schans et al. 2007;Maas et al. 2003). Clinicians tend to hesitate to perform CT-guided lung biopsy in patients with outstanding LAA because they are afraid of severe pneumothorax. TBLB is commonly used in the diagnosis of lung cancer. While navigation systems can increase a diagnostic accuracy for small lesions as early stage lung cancer (Arenberg 2009;Makris et al. 2007;Gildea et al. 2006), it is not accurate enough to replace CT-guided lung biopsy.

It has previously been thought that patients with poor pulmonary functions, especially elderly, had no choice in receiving treatment because they were not thought to be eligible for operation. Ando, et al. previously reported that stereostatic radiotherapy (SRT) is not inferior to surgical operation in the treatment of the elderly with stage I lung cancer (Ando et al. 2010). Nowadays, the patients who cannot receive the operation for early stage lung cancer could have an occasion to treat by SRT with acceptable complication rates (Ando et al. 2010). Moreover, in Japan, mass screening trials for COPD patients to make a diagnosis as lung cancer by CT scan has been ongoing since 2008, that is, it is presumed that more patients with a small lesion will be found and referred to us. Therefore, CT-guided lung biopsy is expected to be performed more frequently for diagnosis of the small lesions as early stage lung cancer. Thus, the indication for CT-guided lung biopsy for patients with emphysema should be established. This is the first report in terms of the relationship between the risk for pneumothorax and COPD or emphysema.

In diagnosing a small lesion as lung cancer, avoiding performing CT-guided lung biopsy for patients with an outstanding emphysema would be reasonable, since clinicians are very afraid of severe pneumothorax that would require chest tube drainage. We suggest that COPD staging of I and II, or high scores of LAA by Goddard classification might not be at risk for pneumothorax. To our surprise, pneumothorax did not occur in patients with higher score of LAA, even though no significant difference in the extent of LAA was seen in the two groups, namely between patients with and without pneumothorax as shown in Table 1. One possible explanation is that operators performed more carefully in patients with outstanding LAA. . For cases with lung fields with LAA scores ≧3, CT-guided lung biopsy was not performed in this study. It might be necessary to investigate the relationship between the Goddard classification of the CT-guided lung biopsy tract and the occurrence of pneumothorax

There are some limitations as follows. First, this is a retrospective study in a very small population. Second, patients with COPD staging of III and IV are not included in this study. Selection bias could possibly exist. Clinically, severe and very severe COPD patients should also be analyzed for the procedure with acceptable tolerability. Third, there might be both interobserver and intraobserver differences in scoring LAA scores.

In conclusion, we suggest that COPD and LAA by Goddard classification may not be related to the frequency of pneumothorax. CT-guided lung biopsy could be performed in patients with COPD staging of I and II with acceptable tolerability. More patients with COPD should be analyzed to make an indication of CT-guided lung biopsy for patients with severe emphysema.
